# *WNT4* Gene and Protein Expression in Endometrial Cancer and Its Significance

**DOI:** 10.3390/cancers15194780

**Published:** 2023-09-28

**Authors:** Jolanta Kiewisz, Tomasz Waśniewski, Jacek Kieżun, Agnieszka Skowrońska, Monika M. Kaczmarek, Błażej Szóstak, Anna E. Kowalczyk, Zbigniew Kmieć

**Affiliations:** 1 Department of Human Histology and Embryology, School of Medicine, Collegium Medicum, University of Warmia and Mazury, 10-082 Olsztyn, Poland; jacek.kiezun@uwm.edu.pl (J.K.); a.kowalczyk@uwm.edu.pl (A.E.K.); zkmiec@gumed.edu.pl (Z.K.); 2 Department of Gynecology and Obstetrics, School of Medicine, Collegium Medicum, University of Warmia and Mazury, 10-082 Olsztyn, Poland; tomasz.wasniewski@uwm.edu.pl; 3 Department of Human Physiology, School of Medicine, Collegium Medicum, University of Warmia and Mazury, 10-082 Olsztyn, Poland; 4 Department of Hormonal Action Mechanisms, Institute of Animal Reproduction and Food Research, Polish Academy of Science, 10-748 Olsztyn, Poland; 5 Department of Pathomorphology, The Regional Specialist Hospital, 10-561 Olsztyn, Poland; 6 Department of Histology, Medical University of Gdansk, 80-211 Gdansk, Poland

**Keywords:** WNT4, estrogen, endometrial cancer, qPCR, IHC

## Abstract

**Simple Summary:**

The dysregulated impact of WNT4 and estrogens disrupts uterine homeostasis and function, potentially escalating the risk of endometrial cancer. This research sought to assess aberrations in *WNT4* gene expression and WNT4 protein immunoreactivity in clinical samples of endometrial cancer, with a focus on tumor characteristics, clinicopathological association, and estrogen dependence. Analysis of a large patient cohort provided additional support for studying *WNT4* expression. Results provide a valuable basis for further investigation of the molecular mechanism of estrogen-induced endometrial carcinogenesis.

**Abstract:**

Background: The inappropriate action of WNT4 and estrogens affects uterine homeostasis and function, and may lead to endometrial cancer (EC). Objective: The aim was to evaluate the alterations of *WNT4* gene expression and WNT4 protein immunoreactivity (Ir) in EC, considering tumor characteristics, the clinicopathological association and estrogen dependence. Methods: *WNT4* mRNA levels were compared between benign (control) endometrium (*n* = 8) and endometroid EC (EEC) and non-endometroid EC (non-EEC) samples (*n* = 28) using the real-time PCR technique. The WNT4-Ir and ERα-Ir were evaluated by immunohistochemistry (IHC). *WNT4* mRNA gene and WNT4-Ir were correlated with clinicopathological and blood morphological parameters. Overall survival (OS) was assessed. The bioanalysis was utilized to study WNT4 expression in large patient cohort (*n* = 549). Results: *WNT4* gene expression was decreased in EC samples (specifically in EEC but not in non-EEC) compared to the control. The *WNT4* gene expression was also decreased in EC samples categorized by the tumor characteristics. There was no statistical difference in WNT4-Ir or ERα-Ir between the control and EC. There was no correlation between OS and *WNT4* gene expression and WNT4-Ir. Bioanalysis showed that *WNT4* and *ESR1* gene expression alterations tended to be mutually exclusive. An alteration in *WNT4* expression was found in different histological tumor types in a large group of EC patients. Conclusions: There is a great need to evaluate the molecular background of EC. Our study suggests that the *WNT4* gene has the potential to be a marker of functional estrogen signaling in EEC.

## 1. Introduction

Carcinoma of the uterine corpus ranks fifteenth among all newly diagnosed cancers in women worldwide. According to the Global Cancer Observatory (GCO), more than 417,000 women developed this disease in 2020. Approximately 97,000 women diagnosed with a uterine tumor died in 2020 (WHO; Global Cancer Observatory; https://gco.iarc.fr/today/data/factsheets/cancers/24-Corpus-uteri-fact-sheet.pdf (accessed on 15 August 2023). Obesity, diabetes, early onset of menarche, nulliparity, late menopause onset, older age (≥55 years), and tamoxifen use are the main risk factors of endometrial cancer (EC) [1,2]. In recent years, there has been a remarkable increase in the EC incidence with aggressive non-endometrioid EC (non-EEC) among Afro-Americans compared to Caucasian women [3]. An increasing number of cases of EC are being detected in women under the age of 40 [4]. However, the etiology of those phenomena has not yet been elucidated. There is an urgent need for further research on the EC incidence in different populations to explain this difference and to explore the molecular factors responsible for this tumor development.

With the latest classification system according to The Cancer Genome Atlas, four molecular subtypes of EC can be distinguished (*POLE* ultramutated, microsatellite instability hypermutated, copy number low, and copy number high [5]). However, ECs have traditionally been divided into two types based on histological features and estrogen dependence. Endometrioid EC (EEC) is an estrogen-dependent adenocarcinoma (Type I [6,7]). The EEC accounts for 80–90% of all EC cases. It is found mainly in premenopausal and perimenopausal women aged 50–65 years [8]. Molecular analysis has shown that mutations associated with EEC are located in *PTEN*, *KRAS*, *CTNNB1*, and DNA repair genes [9]. Serous, clear cell and undifferentiated/dedifferentiated carcinomas, and carcinosarcoma represent an estrogen-independent group of non-EECs which are high grade by convention (Type II) [8]. Serous carcinoma, the prototypical non-EEC, usually occurs in a background of atrophic endometrium of postmenopausal women [8,10] and harbors mutations in the *TP53* gene as well as *ERBB2* and *CDKN2A* gene alterations in a subset of cases [9].

The WNT4 protein is involved in uterine horn formation [11] and gametogenesis during embryonic development [12,13], as well as in oocyte maturation in the adult [14]. Sustained expression of the *WNT4* gene is essential for proper endometrial function during the menstrual cycle [15,16]. In humans, the WNT4 protein is responsible for embryo implantation, and its absence causes recurrent miscarriages [17]. In pathological conditions such as uterine leiomyomas, ovarian tumors, and cervical cancer, *WNT4* gene expression is altered compared to histologically benign tissues [17].

Uterine growth, differentiation and function are under the influence of estrogens and progesterone. Estrogen-mediated gene transcription is controlled by the nuclear estrogen receptors alpha (ERα; *ESR1*) and beta (ERβ; *ESR2*), expressed in both benign and transformed endometrium ([18,19,20]). Changes in the expression of estrogen receptor isoforms are considered markers of uterine tumor progression and patient survival [21]. The mean expression level of ERα protein in EC is 2.9-fold higher than that of ERβ (The Cancer Genome Atlas). High ERα expression fosters EC, whereas ERβ does not [22].

There is evidence that ERα controls *WNT4* gene expression. This has been shown in invasive lobular carcinoma cell lines [23,24] but not in EC. To our knowledge, ERα expression detected by immunohistochemistry (IHC) is elevated in the early stages of EC, whereas it decreases in advanced EC [25]. The original study by Bui and colleagues showed that physiological levels of 17-β-estradiol did not affect *WNT4* mRNA expression [16]. However, these studies were performed without concerning tumor characteristics, with a limited number of patients, cell lines, and experimental replicates. Therefore, our goal is to investigate the *WNT4* gene expression, considering clinical and surgical staging features, as well as WNT4 protein immunoreactivity (Ir) in benign endometrium and EC. We will compare the obtained results with ERα protein Ir. We will correlate WNT4 immunoreactivity (WNT4-Ir) with ERα immunoreactivity (ERα-Ir), and we will correlate blood parameters with *WNT4* mRNA levels and WNT4-Ir in EEC and non-EEC samples. In addition, WNT4 and ERα staining intensities’ semi-quantitative numerical scores will be compared with overall patient survival (OS) to determine whether WNT4 and ERα co-expression is associated with a higher risk of death. Achieving our goal will be a fundamental first step in determining the importance of WNT4 as an oncogenic molecule and determining the mode of interaction between WNTs and estrogen signaling.

## 2. Material and Methods

### 2.1. Patients

#### 2.1.1. Inclusion Criteria

The clinical samples used for molecular studies were previously described [26] (Table S1). Briefly, the cohort consisted of patients from the Division of Gynecology and Obstetrics and Gynecology Oncology Center at The Regional Specialist Hospital in Olsztyn, Faculty of Medicine, University of Warmia and Mazury in Olsztyn, Poland. The control group consisted of 8 patients without endometrial hyperplasia and neoplastic changes (age: 47 ± 7.63, mean ± SD; BMI 18.5–25, *n* = 4; BMI 25–30, *n* = 2; BMI > 30, *n* = 2). Histologically diagnosed EC group, detected by diagnostic curettage of the uterine cavity and treated with surgery (hysterectomy, salpingo-oophorectomy, cytological peritoneal washing and brushing, and pelvic and aortic lymph node dissection), included 28 women (age: 66.95 ± 10.38, mean ± SD; BMI 18.5–25, *n* = 3; BMI 25–30, *n* = 7; BMI > 30, *n* = 18).

#### 2.1.2. Exclusion Criteria

Patients undergoing acute illnesses (i.e., infection, non-infectious inflammation, or cardiovascular events) or disease that had occurred within the last 30 days, known active malignancy, long-term estrogen therapy, polycystic ovarian syndrome, obesity, diabetes mellitus, and hypertension were not qualified for the study. Drug or alcohol abuse excluded patients from the study cohort. All the studied patients did not receive anticancer therapy before surgery.

The requisite clinicopathological information, i.e., age at diagnosis, tumor location, tumor size, and histological grade, was collected by reviewing the patient’s medical records. All procedures were performed following ethical standards and obtained ethical board approval.

### 2.2. Material Collection

Peripheral blood (5 mL) was collected from fastened patients. Blood was centrifuged for 10 min at 3500 rpm. Morphology, Mean Platelet Volume (MPV), and Activated Partial Thromboplastin Time (APTT) were assayed using a Cobas 6000 multianalyzer (Roche Diagnostics, Basel, Switzerland).

From the tumor-suspected area, several samples were preserved to ensure the best homogeneity of the test specimens. Tissue blocks (5 × 5 × 5 mm) were snap-frozen in liquid nitrogen immediately after the tumor was excised. Both control and EC samples were stored at −80 °C for RNA isolation. The tissue was fixed in 10% neutral-buffered formalin for routine diagnostic pathomorphological assessment and IHC evaluation. Fragments were dehydrated in a graded ethanol series (50–96%) and embedded in paraffin for further processing. Histopathological evaluation of hematoxylin and eosin (H&E; Sigma-Aldrich, St Louis, MO, USA) stained sections for tumor phenotype classification was conducted by two qualified pathologists.

### 2.3. RNA Isolation and cDNA Synthesis

Tissue RNA isolation was conducted as described previously [26]. Briefly, all samples were homogenized (MagNA Lyser Instrument and MagNA Lyser Green Beads; Roche Molecular Systems, Inc., Pleasanton, CA, USA), and total RNA was extracted using mirVana Isolation Kit (Thermo Fisher Scientific, Waltham, MA, USA), followed with a spectrophotometric assessment of RNA concentration (NanoDrop 1000; Thermo Fisher Scientific, Waltham, MA, USA). Total RNA (2 µg) was reversibly transcribed using a HighCapacity cDNA Reverse Transcription Kit (Applied Biosystem, Waltham, MA, USA), in line with the manufacturer’s recommendations.

### 2.4. Real-Time PCR

The reactions of *WNT4* and peptidylprolyl isomerase A *(PPIA*) as a reference gene [27] were performed in duplicate and evaluated using real-time PCR, using 7500 Fast Real-Time PCR System (Applied Biosystems, Foster City, CA, USA) and TaqMan assays (Hs01573504_m1 for *WNT4* and Hs99999904_m1 for *PPIA*; Applied Biosystems, Foster City, CA, USA). The thermal cycling conditions were as follows: polymerase activation for 20 s at 95 °C, followed by 40 cycles of denaturation at 95 °C for 3 s, and annealing/extension at 60 °C for 30 s. All samples were quantified in duplicates. No template control reactions were performed for each PCR run. Standard curves consisting of serial dilutions of the appropriate cDNA were used to control the efficiency of qPCR reactions. Relative quantification of *WNT4* expression was evaluated using the ΔCt (dCt) method [28] modified by Pfaffle [29]. Results of the dCt were presented after subtraction from an arbitrary value of 20 so that a high 20-dCt value indicates a high gene expression level.

### 2.5. Immunohistochemistry

Tissue sections for WNT4-Ir and ERα-Ir detection (control (*n* = 7) and EC (*n* = 20)) were deparaffinized, pretreated according to the buffer supplier’s recommendations (Roche Diagnostics, Basel, Switzerland), and transferred to a BenchMark ULTRA IHC/ISH autostainer (Roche Diagnostics, Basel, Switzerland). Slides for ERα-Ir detection (control (*n* = 5) and EC (*n* = 18)) were deparaffinized and treated manually. To detect WNT4-Ir, rabbit anti-human antibodies (Cat. No. HPA011397; Sigma-Aldrich, St Louis, MO, USA) were diluted in NGS/PBS/BSA buffer (1:100). ERα-Ir was detected with rabbit monoclonal antibody (1:1000; Cat. No. ab32063; Abcam, Cambridge, UK). Secondary, biotinylated, goat anti-rabbit antibody (Cat. No. BA-1000; Vector, Newark, CA, USA) was visualized with VECTASTAIN^®^ Elite^®^ ABC-HRP Kit (Cat. No. PK-6102; Vector, Newark, CA, USA) followed by 3, 3-diaminobenzidine (DAB; Sigma-Aldrich, St Louis, MO, USA) as a chromogen. Sections were counterstained with hematoxylin and a coverslipped. Olympus BX-41 light microscope, equipped with an XC 50 camera (Olympus Corporation, Tokyo, Japan) was used to evaluate the slide by an independent scientist, blinded to the patient’s clinical data. The evaluation was randomly verified by an experienced pathologist. Pictures were scanned and archived with Panoramic Digital Slide Scanner MIDI (3DHistech Ltd., Budapest, Hungary). Incubation of sections without primary antibodies was considered a negative control.

### 2.6. The Staining Heterogeneity Evaluation

The Dako procedure of immunoreaction heterogeneity evaluation was described previously for immunophenotypic analysis of inflammatory breast cancers [30] and modified for endometrial-tissue-staining assessment. The evaluation was conducted manually on the same size microscopic fields and under the same magnification (10×) using a scale based on the reaction intensity. A specimen was scored 0 if staining was observed in 10% or less of the cells, regardless of staining intensity. If staining was observed in more than 10% of the cells, the specimen was scored 1+ if barely perceptible intensity staining was observed, 2+ if moderate staining was observed, and 3+ if strong intensity staining was observed.

The evaluation was also confirmed with the free software ImageJ Fiji (version 1.53t), following the protocol of Crowe and Yue [31] for deconvolution of IHC images stained with hematoxylin and DAB. However, the plots are presented to preserve the distribution of staining intensities among patients due to the small number of patients in the study groups.

### 2.7. Database Analysis

The cBioPortal 9 (https://docs.cbioportal.org/ (accessed on 4 September 2023); [32,33]) was utilized to obtain WNT4 expression data, displaying the variability of WNT4 expression in an alternative cohort. The database was queried to retrieve the expression levels of both *WNT4* and *ESR1* genes. The Uterine Corpus Endometrial Carcinoma repository (TCGA, Firehose Legacy; *n* = 549) was chosen from clinicogenomic cancer data for visualization and analysis. Samples were analyzed for mutations, putative copy number alterations from GISTIC, z-score of mRNA expression relative to diploid samples (threshold ±2.0), and z-score of protein expression (threshold ±2.0). Of the 549 repository samples, four hundred forty samples (*n* = 440) were extracted for further analysis.

### 2.8. Statistical Analysis

All statistical analyses were conducted using GraphPad PRISM v. 6.0 software (GraphPad Software, Inc., San Diego, CA, USA). The Mann–Whitney test was used to compare *WNT4* gene expression in the control and all EC samples. The *WNT4* mRNA levels were compared between EEC and non-EEC samples concerning grade, stage, myometrial invasion, and cervical stromal invasion by one-way ANOVA followed by the Kruskal–Wallis post hoc test. The Kruskal–Wallis comparison was used to analyze the differences in WNT4-Ir between the control and EC group, as well as control and endometrial samples of patients diagnosed with EEC and non-EEC. *WNT4* gene and WNT4-Ir correlation with blood parameters in both studied patient groups were calculated using a Spearman test. The one-way ANOVA followed by the Kruskal–Wallis test was also used to compare ERα-Ir between control and EC samples, as well as control and differently graded tumors. Survival curves were plotted and the OS differences were evaluated by a log-rank test for patients followed up for 59 months after surgery. Differences were considered statistically significant at a *p*-value less than 0.05.

## 3. Results

### 3.1. Demographic and Clinical Characteristics of Patients with EEC and Non-EEC

Demographic status, blood biochemical parameters, general tumor characteristics, and status of patients categorized according to the EEC and non-EEC are listed in Table 1.

The EC was classified concerning estrogen dependence (EEC; *n* = 22) and estrogen independence (non-EEC; *n* = 6) determined by pathomorphological evaluation. The International Federation of Gynecology and Obstetrics (FIGO) criteria were used to classify the grade of EC samples, as follows: FIGO grade 1 (G1; *n* = 2), FIGO grade 2 (G2; *n* = 19) and FIGO grade 3 (G3; *n* = 7). Due to the low number of G1 patients, G1 and G2 were grouped together (hereafter named G2). Primary tumor status was described according to the TNM system (T1a+T1b, *n* = 16; T2, *n* = 7; T3a+3b, *n* = 5) following the American Joint Committee on Cancer (AJCC). Depth of myometrium invasion into the inner or outer half (<50%, *n* = 22; >50%, *n* = 6), respectively, absence (0; *n* = 22) or presence (1; *n* = 6) of cervical stromal invasion and presence at the area or beyond the uterus (T1-T2, *n* = 23; T3-T4; *n* = 5) were considered.

### 3.2. WNT4 Gene Expression in EC

The *WNT4* transcripts were found in both control and EC tissue samples. Down-regulation of the gene in a sample was defined as the expression ratio of the *WNT4* gene to the reference gene of <1, while up-regulation was defined as the ratio of >1. The *WNT4* mRNA expression level was decreased in 16/28 (57.14%) and was elevated in 12/28 (42.86%) patients with EC.

**Figure 1:**
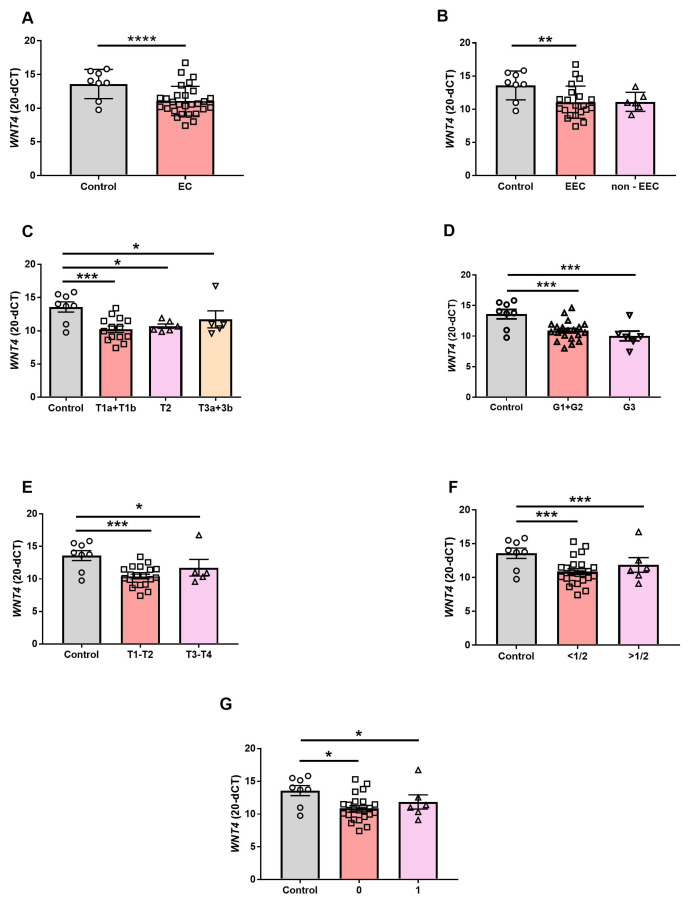
Mann–Whitney test comparison of quantitative real-time PCR result of *WNT4* gene expression in benign endometrium (Control; *n* = 8) and endometrial cancer (EC; *n* = 28) (**A**). Kruskal–Wallis test comparison of the *WNT4* mRNA level in endometrial samples of patients diagnosed with EEC (*n* = 22) and non-EEC (*n* = 6) (**B**). *WNT4* mRNA level in control endometrial samples (*n* = 8) and samples of EC patients depending on the primary tumor status (T1a+T1b, *n* = 16; T2, *n* = 7; T3a+3b, *n* = 5; (**C**)), grade (G1+G2, *n* = 21; G3, *n* = 7; (**D**)), confinement to the uterus (T1-T2, *n* = 23) or extension beyond the uterus (T3–T4; *n* = 5; (**E**)), tumor myometrial invasion (<50%, *n* = 22; >50%, *n* = 6; (**F**)), absence (0; *n* = 22) or presence (1; *n* = 6) of cervical stromal invasion (**G**). Gene expression data were normalized against the *PPIA* mRNA levels and presented as mean 20-dCt ± SEM. The horizontal line shows the mean (* *p* < 0.05; ** *p* < 0.01; *** *p* < 0.001; **** *p* < 0.0001).

Detailed insight into gene expression results revealed that the mean *WNT4* mRNA relative content was reduced in EC patients compared to the control group (*p* < 0.0001; Figure 1A). The *WNT4* transcript level reduction was also observed in the tissue samples of the EEC group (vs. control, *p* < 0.05; Figure 1B), in which the tumor development is estrogen-dependent (*p* > 0.05; Figure 1B). However, there was no difference between control vs. non-EEC (*p* > 0.05; Figure 1B) nor EEC vs. non-EEC groups (*p* > 0.05; Figure 1B).

Furthermore, *WNT4* mRNA expression was significantly reduced in EC samples categorized depending on primary tumor status compared to the control (C vs. T1, *p* < 0.001; C vs. T2, *p* < 0.05; C vs. T3, *p* < 0.05; Figure 1C). There was no difference between groups of different tumor status (T1 vs. T2 vs. T3, *p* > 0.05; Figure 1C). *WNT4* gene expression was also altered in EC samples showing a different malignancy grade only when the level of gene expression was compared to the control (C vs. G1+G2, *p* < 0.001; C vs. G3, *p* < 0.001; Figure 1D). In the group of patients with tumor located within the uterus (T1-T2 group) or tumor extended beyond the uterus (T3-T4 group); *WNT4* mRNA gene expression was lower when compared to control (*p* < 0.001, *p* < 0.05, respectively; Figure 1E). However, the level of *WNT4* gene expression was similar in the T1-T2 and T3-T4 groups (Figure 1E).

Comparison of control tissues to the tissues of the patients with inner half of the myometrium occupied by the tumor or to the tissue of patients where the tumor invades the outer half of the myometrium showed decreased abundance of the *WNT4* transcripts in tumor groups (C vs. <50%, *p* < 0.001; C vs. >50%, *p* < 0.001; Figure 1F). No difference was observed in samples collected when the tumor occupied the inner half of the myometrium in comparison to samples collected when the tumor extended beyond half of the myometrium (<50% vs. >50%, *p* > 0.05; Figure 1F).

Moreover, the reduction of *WNT4* gene expression in EC samples was observed independently from the absence or presence of cervical stromal invasion (C vs. 0, *p *< 0.05; C vs. 1, *p *< 0.05; Figure 1G). There was no difference between groups without and with cervical stromal invasion (0 vs. 1, *p* > 0.05; Figure 1G).

### 3.3. The WNT4-Ir in EC

Benign endometrium and EC presented WNT4-Ir in the cytoplasm of the surface and glandular epithelial cells but not in the endometrial stromal cells.

**Figure 2:**
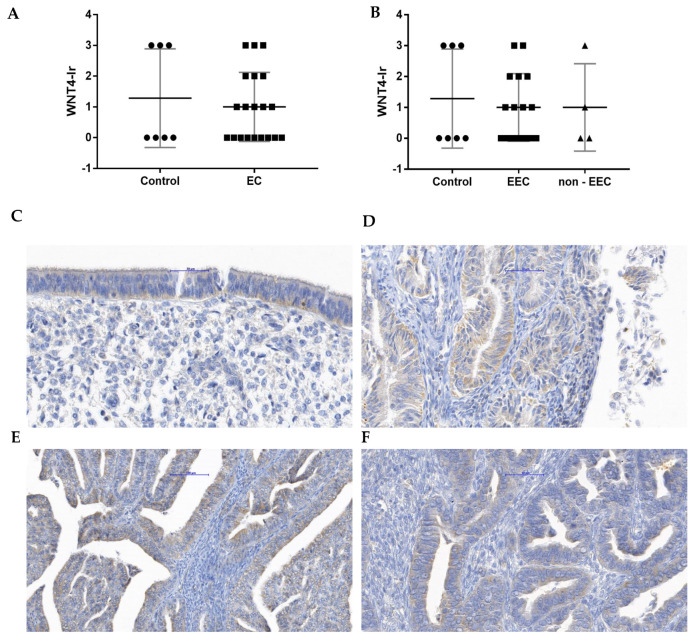
The Kruskal–Wallis comparison of WNT4-Ir between control (*n* = 7) and EC group (*n* = 20; (**A**)) as well as control (*n* = 7) and endometrial samples of patients diagnosed with EEC and non-EEC (EEC, *n* = 16; non-EEC *n* = 4; (**B**)). For each graph, the horizontal line shows the mean with SD. The representative microphotographs present WNT4-Ir in benign endometrium (**C**) and endometrial tissue fragments from samples where tumors were graded as G1 (**D**), G2 (**E**), and G3 (**F**). Magnification ×40.

No difference was observed when WNT4-Ir was compared between the control and EC group (Figure 2A) as well as EEC and non-EEC (Figure 2B) groups. Representative microphotographs showing WNT4-Ir localization in unchanged endometrium (Figure 2C) and endometrial tumors graded as G1 (Figure 2D), G2 (Figure 2E) and G3 (Figure 2F) are presented in Figure 2.

### 3.4. Blood Biochemical Parameters Correlation with WNT4 Gene Expression and WNT4-Ir in EC

The level of *WNT4* mRNA in EC tissue was compared to the biochemical parameters of patients’ blood (Table 2). The only correlation found was between WNT4-Ir and Activated Partial Thromboplastin Time (APTT) in the EEC group (*p* < 0.05).

### 3.5. The Comparison of WNT4-Ir and ERα-Ir in Control and EC Samples

**Figure 3:**
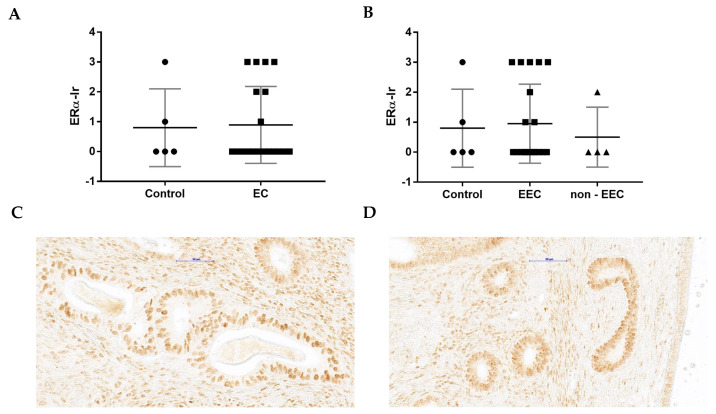
The Kruskal–Wallis test comparison of ERα-Ir between control (*n* = 5) and EC group (*n* = 18; (**A**)) as well as control (*n* = 5) and differently graded tumors (EEC *n* = 14, non-EEC *n* = 4; (**B**)). For each graph, the horizontal line shows the mean with SD. The representative microphotographs presenting ERα-Ir in benign endometrium (**C**,**D**). The images are from slides that were not H&E-stained, giving a better visualization of nuclear staining. Magnification ×40.

ERα-Ir did not differ between EC group (Figure 3A) and control (Figure 3C,D). Moreover, the same tendency was observed for EEC or non-EEC groups (Figure 3B).

A comparison of WNT4-Ir and ERα-Ir in the EC slide from the same patient revealed five cases where both WNT4-Ir and ERα-Ir were not present. In seven samples, only WNT4-Ir was observed, while in two, only ERα-Ir. The simultaneous presence of WNT4-Ir and ERα-Ir on the same slide was observed in only four patients with EC (Table 3).

### 3.6. WNT4 mRNA Level and WNT4-Ir Correlation with OS of EC Patients

The *WNT4* mRNA level and WNT4-Ir results were used to evaluate the usefulness of WNT4 expression as a prognostic factor. All patients (*n* = 28) were followed up for a median time of 59 months (range: 5–78 months). Twenty-five percent (*n* = 7) of patients died during the follow-up period due to EC.

**Figure 4:**
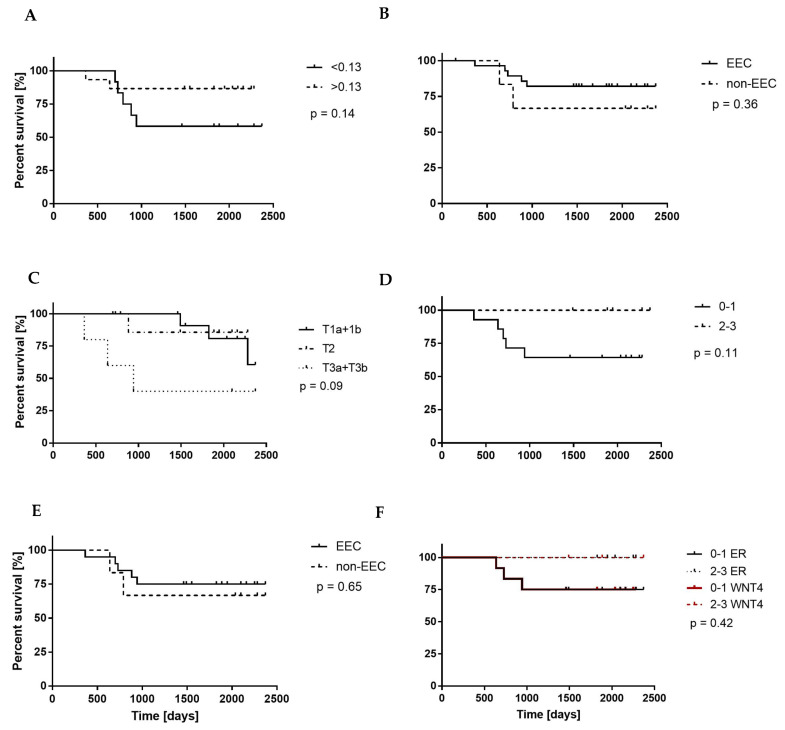
Kaplan–Meier overall survival (OS) curves of the patients with endometrial cancer (EC) in relation to (**A**) the median value of the *WNT4* mRNA level in EC samples, (**B**) the *WNT4* mRNA level in endometrial samples of patients diagnosed with EEC and non-EEC, (**C**) *WNT4* mRNA level in samples of EC classified depending on the primary tumor status. The OS curves in relation to (**D**) the presence of WNT4-Ir in all EC samples and (**E**) WNT4-Ir in tumors classified depending on diagnosis (EEC or non-EEC) are also presented. The (**F**) graph compared the OS curves in relation to the intensity of WNT4-Ir and ERα-Ir in all EC samples. *p*-values of the log-rank test are shown.

The median value of *WNT4* mRNA level (RQ = 0.13; *p* = 0.14; Figure 4A) and WNT4-Ir divided into weak (0–1) and strong (2–3) staining intensity in all EC samples (*p* = 0.11; Figure 4D), *WNT4* mRNA level (*p* = 0.36; Figure 4B) and WNT4-Ir (*p* = 0.65; Figure 4E) in endometrial samples of patients diagnosed with EEC and non-EEC, and *WNT4* gene expression in EC samples classified depending on primary tumor status (*p* = 0.09; Figure 4C) did not correlate with patients’ OS. Although WNT4-Ir and ERα-Ir in samples exhibiting weak (0–1) and strong (2–3) immunostaining did not correlate with OS (*p* = 0.42; Figure 4F), among patients evaluated in this study, only those exhibiting both weak (0–1) WNT4-Ir and ERα-Ir died.

### 3.7. WNT4 Expression Diversity in a Large Patient Cohort

**Figure 5:**
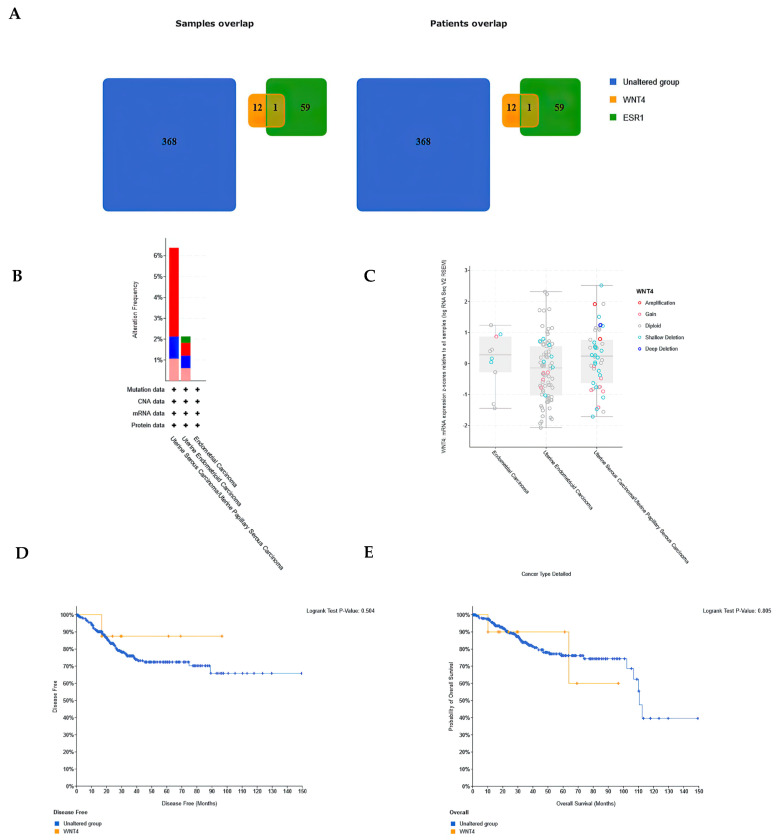
Comparison of *WNT4* gene expression in a patient cohort from the Uterine Corpus Endometrial Carcinoma database in the TCGA repository. (**A**) Groups with altered *WNT4* and *ESR1* gene expression overlapped. Colors denote selected groups, and numbers indicate the corresponding number of patients. Groups that exhibited altered expressions of both genes (*WNT4* and *ESR1*) overlapped. (**B**) Histogram of the frequency of changes in *WNT4* gene expression for each specific cancer type (green—mutations, red—gene amplification, blue—deep deletion, pink—high mRNA). (**C**) Comparison of clinical characteristics with *WNT4* gene expression z-scores relative to all samples. Disease-free status (DFS; (**D**)) and overall survival (OS; (**E**)) curves of patients with unchanged and altered gene expression.

The analysis of the Uterine Corpus Endometrial Carcinoma database in the TCGA repository showed that the *WNT4* gene was altered in 13 (3%) samples, while *ESR1* was altered in 60 (14%) of 440 queried patient profiles (Supplementary Figure S1). Notably, of the 13 samples with altered *WNT4* gene expression, 12 showed no alteration in *ESR1* gene expression. Conversely, out of 60 samples with altered *ESR1* gene expression, only 1 possessed altered *WNT4* gene expression (Figure 5A). The *WNT4* and *ESR1* gene expression alterations tended to be mutually exclusive (Supplementary Table S2). The alteration in *WNT4* gene expression tended to be more frequent in serous carcinomas (6 of 94 cases (6.38%); 4 cases—gene amplification; 1 case—deep deletion; 1 case—high mRNA; Uterine Serous Carcinoma/Uterine Papillary Serous Carcinoma Subtypes) than in EEC (7 of 329 (2.13%) cases; 1 case—mutation; 2 cases—gene amplification; 2 cases—deep deletion; 2—high mRNA; Uterine Endometrioid Carcinoma Subtypes) (Figure 5B). Plots of expression of *WNT4* genes comparing clinical attributes to the mRNA expression z-scores relative to all samples (log RNA Seq V2 RSEM) are presented in Figure 5C. Patients with alterations in *WNT4* gene expression compared with patients with unaltered gene expression presented no difference in disease free status since initial treatment (log-rank test *p* value 0.315; Figure 5D) and OS status (log-rank test *p* value 0.949; Figure 5E).

## 4. Discussion

Consistent with the hallmark study of Bui [16] and the recent study of Coopes [34], we showed that *WNT4* gene expression was significantly decreased in EC compared to the control. Furthermore, our study is consistent with the data showing that *WNT4* is a down-regulated gene in EC, which was detected using microarray [35] and next-generation transcriptome sequencing technology [36]. A novelty of our approach is the comparison of *WNT4* gene expression between control and EC samples, taking into account not only grade but also other cancer features. In addition, we are the first to show that the reduction in *WNT4* mRNA levels is mainly observed in estrogen-dependent EC samples.

In contrast, high *WNT4* gene expression has been reported in human breast cancer tissue [37], thymoma tissue [38], and colorectal cancer [39]. An increase in *WNT4* gene expression has also been detected by quantitative real-time PCR in benign gynecological lesions such as leiomyomas [40], in human papillomavirus-associated cervical cancer [41] and by real-time PCR in ovarian tumors [42,43]. The discrepancies in expression levels may be because WNT4 plays context-dependent roles depending on intra- and extracellular activity [17], activated receptors, and signaling pathways [44].

We observed decreased *WNT4* gene expression in estrogen-dependent EEC but not in non-EEC tissues compared to the control samples. It is well known that the predominance of estrogen activity can trigger the development of EC [45]. In addition, a complex of estrogen and ERα acts as a transcription factor that modulates the expression of *WNT4* [46]. Therefore, we might have expected an increased expression of ERα during the development of EEC. The *WNT4* mRNA levels may also have increased in EEC compared to the control. However, our data do not support such a hypothesis. Estrogens modulate the expression of *WNT4* gene [41], but this may be due to the fact that they can activate the transcription of target genes not only through direct estrogen–receptor complex binding to estrogen response elements at the DNA level but also through physical interaction with other factors [47]. Moreover, *WNT4* and *ESR1* gene expression alterations tended to be mutually exclusive, which is what we confirmed in our study with the bioinformatic analysis. Thus, while the tumor-promoting effects of estrogens can be maintained, the activation of *WNT4* gene expression can be neglected. The presence of a specific *WNT4* allele variant (rs3820282) has been shown to alter ER activity and increase estrogen-mediated *WNT4* expression [46]. Thus, the lack of rs3820282 allele variant could also explain the reduced expression of the *WNT4* gene in the studied EC group. If this is the case in our study group, it would require further investigation, since such alterations combined with mutations are rare as evidenced by our cohort analysis with the cBioPortal platform (1 case per 329 patients whose data were deposited in the Uterine Endometrioid Carcinoma repository).

Our study demonstrates a decrease in *WNT4* gene expression in estrogen-dependent EEC, which suggests the potential for the *WNT4* gene as a marker of functional estrogen signaling in EEC. This finding aligns with previous research showing a negative effect of estradiol on *WNT4* gene expression under in vitro conditions [48]. The study also agrees with the observation that the expression of the *WNT4* gene, along with the action of WNT4 glycoprotein, is directly regulated by estrogen in invasive lobular breast carcinoma cell lines [23]. Indirectly, the hypothesis is supported by the results, which demonstrate a negative correlation between *WNT4* gene expression and estradiol content in uterine lumen fluids on day 12 of gestation and elevated *WNT4* gene expression in the endometrium with reduced estradiol content in the uterine lumen fluids of gilts with hormonally synchronized estrus [49]. By designating *WNT4* as a potential marker of functional estrogen signaling, our study strengthens the notion that estradiol is the main factor responsible for endometrial carcinogenesis. At the same time, a cohort analysis utilizing the cBioPortal platform indicates that *WNT4* might be significant for the growth of various histological tumor types as among a large group of EC patients, an alteration in *WNT4* expression was found to be evident within the serous carcinoma. We did not have any statistical confirmation of the differences in WNT4-Ir or ERα-Ir between the study groups. Interestingly, we show a statistically significant correlation between APTT and WNT4-Ir in EEC patients. There is scarce information regarding the APTT’s significance in the process of endometrial carcinogenesis. The prognostic value of the APTT has not been confirmed in EC [50]. The inverse correlation between WNT4-Ir and APTT levels suggests that down-regulation of *WNT4* gene expression may activate coagulation processes and increase the intensity of fibrinogen-to-fibrin conversion. The morphological particles involved in the fibrinogen-to-fibrin conversion are platelets [51]. Their activation and aggregation are associated with the metastatic potential of tumor cells [52]. Whether this plays a role in EC metastasis requires further investigation.

The *WNT4* gene expression and WNT 4–Ir in EC did not show a statistically confirmed WNT4 influence on patients’ OS. It is consistent with what we revealed with our cohort analysis with the cBioPortal platform and the data presented in the Human Protein Atlas (https://www.proteinatlas.org/ (accessed on 4 September 2023) [22]). The WNT4 protein can be considered a favorable prognostic marker in pancreatic cancer and an unfavorable prognostic marker in melanoma, but not in the pathologically altered uterus or other reproductive organs [22]. However, it should be noted that in our study, patients who died had low levels of WNT4 and ERα-Ir.

We are aware that our research may have limitations. The first is the limited number of non-EEC patients. The selected patients were characterized in detail for proper classification. However, it is difficult to recruit a remarkable number of patients in a non-EEC group, especially when cases of estrogen-independent cancers are less common. Second, WNT4-Ir and ERα-Ir comparison was not performed using the double-staining method on the same tissue sections.

## 5. Conclusions

To the best of our knowledge, this is the first comprehensive *WNT4* gene expression analysis in EC patients when tumor-related features are categorized according to status, grade, myometrial infiltration, or location beyond the uterus. The results of our study suggest that the WNT4 protein cannot be directly attributed to tumor invasion and development, but the *WNT4* gene has the potential to be a marker of functional estrogen signaling in EEC. The presentation of *WNT4* gene expression concerning clinical and surgical staging features, alongside WNT4-Ir in benign endometrium and EC, offers a valuable foundation for further inquiries into the molecular mechanism of estrogen-induced endometrial carcinogenesis. Further studies utilizing in vitro and in vivo models will be crucial to comprehend the regulation of WNT4 protein expression via estrogen-activated estrogen receptors. Learning about the influence of the WNT4 protein on intracellular processes, such as metabolism regulation, tumor cell proliferation, and the formation of tumor blood vessels, will enable subsequent studies of tumor reemission, metastasis, and chemoresistance. In the long term, this could lead to the establishment of a more patient-specific risk profile and the development of new personalized therapies.

## Figures and Tables

**Table 1:** Demographic status, blood biochemical and general characteristics of patients with endometrial cancer (EC) group (*n*  =  28).

	Characteristics	Units	EC	
			EEC	non-EEC
	Total number of patients	*-*	22	6
Demographical status	Age	years	65.04 ± 10.25	69.5 ± 9.73
	BMI	kg/m	32.35 ± 5.19	27.88 ± 5.43
Blood biochemical characteristic	RBC	10^6^/μL	4.8 ± 0.43	4.68 ± 0.52
	Hb	g/dL	13.9 ± 1.43	13.7 ± 1.13
	Ht	%	42.1 ± 3.25	41.08 ± 3.01
	WBC	10^3^/μL	7.57 ± 2.19	6.62 ± 1.94
	Granulocytes	%	59.95 ± 8.64	61.68 ± 9.76
	Lymphocytes	%	30.81 ± 7.74	29.15 ± 8.72
	Monocytes	%	6.68 ± 2.05	7.00 ± 1.67
	Platelets	10^3^/μL	233.5 ± 61.59	216.5 ± 58.31
	MPV	fl	11.06 ± 0.75	10.45 ± 0.75
	APTT	s	26.77 ± 2.15	30.15 ± 6.35
General characteristics	Histological subtypes	G1	2	0
		G2	16	3
		G3	4	3
	FIGO stage	T1	13	3
		T2	7	0
		T3	2	3
		T4	0	0
	Vital status	Alive	17	4
		Dead	5	2

Results are presented as Mean ± SD. Abbreviations: APTT—Activated Partial Thromboplastin Time; BMI—Body Mass Index; EEC—endometrioid endometrial cancer; FIGO—International Federation of Gynecology and Obstetrics; Hb—hemoglobin; Ht—hematocrit; MPV—Mean Platelet Volume; n/a—not applicable; non-EEC—non-endometrioid endometrial cancer; RBC—Red Blood Cells; WBC—White Blood Cells.

**Table 2:** Correlations (r) between blood parameters and *WNT4* mRNA levels or WNT4-Ir in EEC and non-EEC group.

	*WNT4* mRNA		WNT4-Ir	
	EEC	non-EEC	EEC	non-EEC
RBC	−0.14	−0.24	0.06	−0.40
Hb	−0.37	−0.16	0.41	−0.20
Ht	−0.43	−0.12	0.49	−0.20
WBC	−0.48	0.24	−0.38	0.40
Granulocytes	−0.03	0.31	0.23	0.80
Lymphocytes	0.37	−0.32	−0.28	−0.80
Monocytes	0.83	0.06	−0.13	−1
Platelets	−0.6	0.17	−0.25	−0.20
MPV	−0.14	0.39	0.23	0.40
APTT	0.37	−0.06	−0.51 *	−0.80

Abbreviations: APTT—Activated Partial Thromboplastin Time; BMI—Body Mass Index; EEC—endometrioid endometrial cancer; FIGO—International Federation of Gynecology and Obstetrics; Hb—hemoglobin; Ht—hematocrit; MPV—Mean Platelet Volume; non-EEC—non-endometrioid endometrial cancer; RBC—Red Blood Cells; WBC—White Blood Cells; Statistical significance is bolded and marked as * *p* < 0.05.

**Table 3:** Paired comparison of WNT4-Ir and ERα-Ir in EC slices.

No.	WNT4-Ir	ERα-Ir	SURVIVAL (0/1)
1	0	0	0
2	0	0	1
3	0	0	0
4	0	0	0
5	0	0	0
6	1	0	1
7	1	0	1
8	1	0	0
9	2	0	0
10	3	0	0
11	3	0	0
12	3	0	0
13	0	2	0
14	0	3	0
15	2	2	0
16	1	3	0
17	1	3	0
18	2	3	0

The covered area of the Ir regardless of its intensity: 0—reaction in 10 or less percent (%) of the cells; 1—reaction in more than 10% of the cells but less than 40%; 2—moderate reaction (40–60%), 3—strong reaction (60% or more). Protein overexpression was considered as 2+ and 3+. Survival: 0—alive; 1—died.

## Data Availability

The datasets used and analyzed during the current study are available from the corresponding author on reasonable request.

## Supplementary Materials

The following supporting information can be downloaded at: https://www.mdpi.com/article/10.3390/cancers15194780/s1, Table S1. Detailed patient characteristics; Table S2: Mutual exclusivity of alteration of *WNT4* and *ESR1* gene; Figure S1. Alteration in the expression of *WNT4* and *ESR1* genes observed in samples from the Uterine Corpus Endometrial Carcinoma database in the TCGA repository.
