# Supplementary material for: WNT4 Gene and Protein Expression in Endometrial Cancer and Its Significance
Source: Cancers (Basel). 2023 Sep 28;15(19):4780. doi: 10.3390/cancers15194780 (PMC10571897; doi:10.3390/cancers15194780)
Supplement: Supplementary file 1 [file cancers-15-04780-s001.zip › Supplementary Table S2.pdf]

Supplementary Table S2. Mutual exclusivity of alteration of *WNT4* and *ESR1* gene

A	B	Neither A Not B	Neither B Not A	Neither Both	Log2	Odds Ratio	p-Value	q-Value	Tendency
WNT4	ESR1	35	4	6	0	<-3	1.000	1.000	Mutual exclusivity
