# Supplementary figures and images for: WNT4 Gene and Protein Expression in Endometrial Cancer and Its Significance
Source: Cancers (Basel). 2023 Sep 28;15(19):4780. doi: 10.3390/cancers15194780 (PMC10571897; doi:10.3390/cancers15194780)

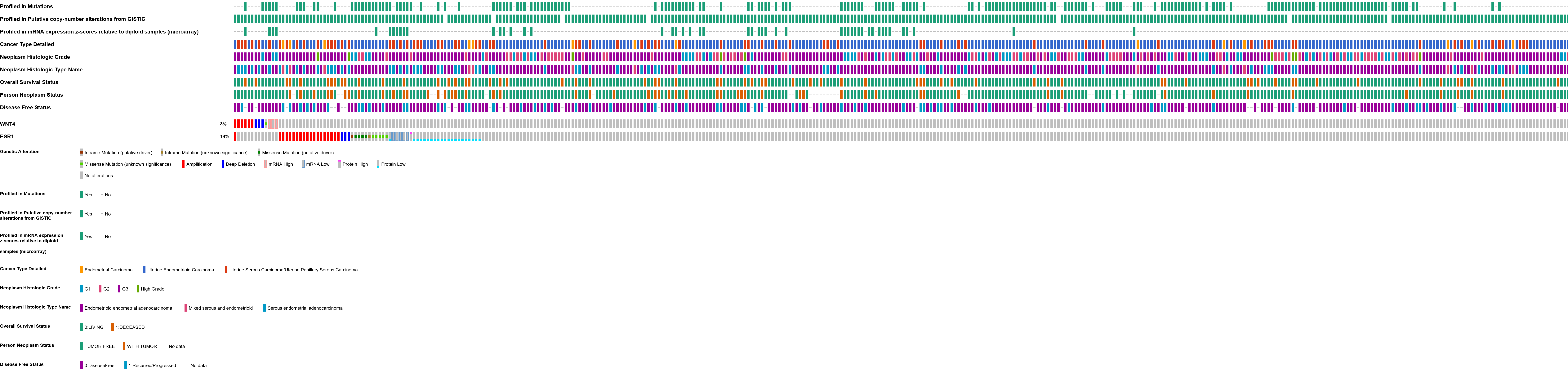

Supplement: Supplementary file 1 [file cancers-15-04780-s001.zip › Supplementary Figure S1.png]
